# HPV-16 E6 and E7 Oncoproteins Promote Cell Proliferation and Migration Through the miR-218-5p/*PIK3C2A* Axis

**DOI:** 10.3390/pathogens15060648

**Published:** 2026-06-19

**Authors:** Brayan Villazana-Calderon, Hilda Jiménez-Wences, J. Noé García-Chávez, Imelda Martínez-Ramírez, Claudia González-Espinosa, Gloria Fernández-Tilapa, Marcela Lizano, J. Omar Muñoz-Bello

**Affiliations:** 1 Subdirección de Investigación Básica, Instituto Nacional de Cancerología, Mexico City 14080, Mexico; bvillazanac@gmail.com (B.V.-C.); imartinezr@incan.edu.mx (I.M.-R.); 2 Facultad de Ciencias Químico-Biológicas, Universidad Autónoma de Guerrero, Chilpancingo 39090, Mexico; hjimenez@uagro.mx (H.J.-W.); gfernandezt@uagro.mx (G.F.-T.); 3 Tecnológico Nacional de México/ITS de Irapuato, Irapuato 36821, Mexico; j.noe.garcia.c@gmail.com; 4 Departamento de Farmacobiología, Centro de Investigación y de Estudios Avanzados, Unidad Sede Sur, Mexico City 14330, Mexico; cgonzal@cinvestav.mx; 5 Centro de Investigación Sobre el Envejecimiento, Centro de Investigación y de Estudios Avanzados, Unidad Sede Sur, Mexico City 14330, Mexico; 6 Departamento de Medicina Genómica y Toxicología Ambiental, Instituto de Investigaciones Biomédicas, Universidad Nacional Autónoma de México, Ciudad Universitaria, Mexico City 04510, Mexico

**Keywords:** HPV oncoproteins, E6 and E7, *PIK3C2A*, miR-218-5p, cervical cancer

## Abstract

The continuous expression of HPV oncogenes E6 and E7 contributes to the maintenance of the cervical cancer (CC) phenotype by altering gene expression programs involved in tumor progression and aggressiveness. MicroRNAs (miRNAs) have emerged as critical regulators of gene expression in CC, including miR-218-5p, which has been described as a tumor suppressor. In this study, we investigated the impact of HPV-16 oncoproteins E6 and E7 on the regulation of miR-218-5p expression and its target gene *PIK3C2A*, as well as their functional and clinical relevance in CC. We found that miR-218-5p expression is significantly reduced in HPV-16-positive CC cell lines, while *PIK3C2A* expression is increased. Silencing the expression of the E6/E7 oncogenes in Ca Ski cells restored miR-218-5p levels and reduced *PIK3C2A* expression. Conversely, overexpression of the E6 and E7 oncogenes in C-33 A cells significantly decreased miR-218-5p expression and increased *PIK3C2A* expression. Functional assays performed on C-33 A cells expressing E6 and E7 revealed that ectopic expression of miR-218-5p suppresses cell proliferation and migration, effects that are partially mediated by *PIK3C2A*. Bioinformatics analysis showed that low miR-218-5p expression and high *PIK3C2A* expression are associated with reduced overall survival in patients with cervical cancer. Our findings identify the miR-218-5p/*PIK3C2A* axis as a novel regulatory pathway modulated by HPV-16 oncoproteins E6 and E7 that contributes to CC cell proliferation and migration. Furthermore, miR-218-5p and *PIK3C2A* emerge as potential prognostic biomarkers in CC.

## 1. Introduction

Cervical cancer (CC) is the fourth leading cause of cancer-related death among women worldwide, with an estimated 348,874 deaths reported in 2022 [1]. The primary etiological factor in the development of CC is persistent infections with high-risk human papillomavirus (HPV) types [2]. Approximately thirteen high-risk HPV types can infect the anogenital epithelium, typically resulting in transient infections that are effectively controlled by the host immune system [3]. Most high-risk HPV infections are cleared within 12 to 18 months; however, approximately 10% of women fail to eliminate the virus, leading to long-term persistent infections. Persistence high-risk HPV infections beyond 18 months, represent the most significant risk factor for the development of cancer [4]. In CC, the most prevalent high-risk HPV types are types 16 and 18, which account for approximately 70% of all cases [5]. The oncogenic potential of high-risk HPV is attributed to the activity of the E6 and E7 oncoproteins, whose sustained expression promotes cancer-related cellular processes, such as migration, invasion, and exacerbated cell proliferation [6,7]. Specifically, HPV oncoproteins can interact with a wide variety of cellular proteins, altering their function. For instance, E6 interacts with p53, promoting its degradation dependent on the ubiquitin ligase E6AP [8]. Meanwhile, the E7 oncoprotein promotes pRb degradation dependent on the ubiquitin ligase Cullin 2 [9]. These events promote apoptosis evasion, mutation accumulation, and cell cycle continuity [10].

Previously, multiple cell signaling pathways have been found to be altered by HPV oncoproteins, including Wnt/β-catenin [11,12], Notch [13], and PI3K/Akt [14], among others [15]. The phosphatidylinositol 3-kinase (PI3K)/Akt pathway is aberrantly active in several cancer types, including HPV-related cancers, contributing to tumor development and progression [16,17]. PI3K is known to phosphorylate phosphatidylinositol-4,5-bisphosphate (PI(2,5)P2) to generate phosphatidylinositol-3,4,5-trisphosphate (PI(3,4,5)P3), an important molecule for the recruitment of signaling proteins to the plasma membrane such as Akt, a threonine serine kinase that is a central node in several signaling pathways as it phosphorylates multiple downstream substrates such as mTOR, thereby affecting cell survival [16].

Three classes of PI3K have been identified (Class I, II, and III), each with unique preferences for phosphoinositide substrates and producing specific lipid second messengers, responding to a wide variety of signaling molecules. Class II consists of three members including PI3K-C2α, PI3K-C2β, and PI3K-C2γ, these kinases are structurally composed of a Ras-binding domain and a PI3K catalytic domain but lack a regulatory subunit. They seem to be high molecular weight monomers, predominantly associated with intracellular membranes [18]. Lipid products generated from Class II PI3 kinases include phosphatidylinositol 3-phosphate (PI3P) and phosphatidylinositol (3,4)-bisphosphate (PI(3,4)P2). Furthermore, PI3K-C2α, which is encoded by *PIK3C2A* gene, in addition to producing PI3P, also generates PI(3,4)P2 and phosphatidylinositol (3,4,5)-trisphosphate (PI(3,4,5)P3), albeit in low amounts [19]. Class II PI3Ks are activated downstream of several types of receptors, including tyrosine kinases receptors such as EGFR and PDGFR [18,20]. Furthermore, PI3K-C2α is known to regulate clathrin-mediated endocytosis, vesicular trafficking, and mitosis. The catalytic activity of PI3K-C2α is associated with the subsequent activation of downstream elements, such as Rab11, RhoA and Akt, affecting several cellular processes [21]. Previous studies have shown that PI3K-C2α is altered in cancer. For instance, in hepatocellular carcinoma cells, miR-26a was found to bind to *PIK3C2A* mRNA, causing its degradation and preventing angiogenesis through the PI3K-C2α/Akt/HIF-1α/VEGFA pathway [22]. In addition, another study found that PI3K-C2α ablation affected cell growth and significantly reduced the colony-forming ability of hepatocellular carcinoma-derived cells [23]. Furthermore, PI3K-C2α has been shown to be required for genomic stability, as its downregulation leads to spindle abnormalities, delayed anaphase onset, and aneuploidy. In breast cancer mice models, low levels of PI3K-C2α initially hamper tumor development but subsequently accelerate cancer growth with mitotic checkpoint defects. Loss of PI3K-C2α increases sensitivity to taxane-based therapy in preclinical models and neoadjuvant settings [24]. However, the involvement of PI3K-C2α in cervical carcinogenesis or CC progression has not been described so far.

Previous studies in CC-derived cell lines demonstrated that P3KC2A transcript is a target of miR-218-5p, miR-23b-3p, and miR-124-3p [25,26], and that high *PIK3C2A* expression is associated with worse survival in patients with CC [26]. Interestingly, studies carried out in chondrocytes demonstrated that *PIK3C2A* mRNA is a target of miR-218-5p, and that downregulation of miR-218-5p increased *PIK3C2A* expression and induced PI3K/Akt/mTOR pathway signaling [27]. Moreover, exogenous expression of miR-218-5p has been shown to significantly reduce proliferation and induce apoptosis in CC-derived cell lines [28]. Furthermore, miR-218-1 expression decreased in CC compared to premalignant samples. Similarly, miR-218-1 promoter methylation levels are increased in CC and decreased in premalignant lesions [29]. Bioinformatics studies reveal that miR-218-5p expression is decreased in cancer compared to normal tissue, and that low miR-218-5p expression is associated with a worse prognosis [30]. To date, the involvement of the HPV-16 E6 and E7 oncoproteins in the regulation of the miR-218-5p/*PIK3C2A* axis during cervical cancer progression has not been investigated. Therefore, this study aimed to determine the effect of HPV-16 E6 and E7 on miR-218-5p and *PIK3C2A* expression and to assess the consequences of this regulation on the proliferative and migratory capacities of cervical cancer cells, two hallmarks associated with tumor progression and aggressiveness.

## 2. Materials and Methods

### 2.1. Cell Lines and Culture

CC cell lines C-33 A (HTB-31), Ca Ski (CRL-1550) and SiHa (HTB-35) were purchased from American Type Culture Collection (ATCC). In addition, C-33 A cell lines were stably transfected with the expression vector p3XFLAG-CMV™-10 (Sigma Aldrich, St. Louis, MO, USA) and with constructs generated in the same vector containing the coding sequences of the HPV-16 E6 and E7 oncogenes tagged with a hemagglutinin (HA) at the N-terminus, as previously described [31]. These C-33 A cell lines were named in our study as EV (empty vector), E616, and E716, which were grown in Dulbecco’s modified Eagle’s medium (DMEM) supplemented with 2 g/L G418 (Santa Cruz Biotechnology, Dallas, TX, USA). Ca Ski cells were grown in Roswell Park Memorial Institute (RPMI) medium; meanwhile, C-33 A and SiHa cell lines were grown in DMEM. All media were supplemented with 10% fetal bovine serum (FBS), and cultures were maintained at 37 °C in a 5% CO_2_ atmosphere.

### 2.2. siRNAs, miRNA Mimics and Plasmids Transfection

For silencing of E6 and E7 HPV-16 gene expression, Ca Ski cell line was transfected with 100 pmol of small interfering RNAs (siRNAs) targeting HPV-16 E6 and E7 (Santa Cruz Biotechnology, Dallas, TX, USA) or with 100 pmol of luciferase siRNAs as a control (Dharmacon, Lafayette, CO, USA). Transfections were performed using Lipofectamine RNAiMAX reagent (Invitrogen, Carlsbad, CA, USA) according to manufacturer’s instructions. Seventy-two hours post-transfection, the cells were lysed for RNA and protein extraction.

For miRNA mimic transfection, C-33 A EV, E616, and E716 cells were transfected with 100 nM of hsa-miR-218-5p mimic (Assay ID: MC10328) or negative control (Scrambled sequence) (Ambion, Austin, TX, USA) using Lipofectamine RNAiMAX reagent (Invitrogen, Carlsbad, CA, USA), following the manufacturer’s protocol. Cells were harvested 24 h post-transfection to perform functional assays, and at 48 h for RNA or protein extraction.

GFP-*PIK3C2A* expressing plasmid was kindly provided by David Bryant and Volker Haucke (Addgene_161988) [32]. C-33 A EV and E7 cells were transfected with 4 µg of GFP-*PIK3C2A* plasmid or the empty vector control pCDNA3.1 (Invitrogen, Carlsbad, CA, USA) using Lipofectamine 2000 reagent (Invitrogen, Carlsbad, CA, USA), according to the manufacturer’s instructions. After 6 h, the medium was replaced, and the cells were subsequently transfected with the hsa-miR-218-5p mimic or scrambled control using Lipofectamine RNAiMAX. Twenty-four hours later, cells were collected for proliferation and migration assays.

### 2.3. Gene Expression Assay by Real-Time Quantitative PCR

A total of 500,000 cells were seeded in 60 mm culture dishes and, when indicated, miRNA was isolated using the miRNeasy Micro Kit (Qiagen, Hilden, Germany), while mRNA was extracted using the RNeasy Micro Kit (Qiagen, Hilden, Germany), following the manufacturer’s instructions in both cases. Samples were treated with the DNA-free kit (Applied Biosystems, Foster City, CA, USA) to eliminate residual genomic DNA. RNA quantification and purity was determined using the NanoDrop One spectrophotometer (ThermoScientific, Waltham, MA, USA).

For miR-218-5p expression analysis, reverse transcription was performed using the TaqMan microRNA Reverse Transcription Kit (Applied Biosystems, Foster City, CA, USA), following the manufacturer’s instructions. Quantitative PCR (qPCR) was carried out using the TaqMan hsa-miR-218-5p probes (Assay ID: 000521) (Applied Biosystems). The miRNA expression levels were normalized to RNU6 (U6 snRNA, Assay ID: 001973) (Applied Biosystems, Foster City, CA, USA) as an endogenous control. To assess the expression of the *PIK3C2A*, E6, and E7 genes, reverse transcription was performed using the High-Capacity cDNA Reverse Transcription Kit (Applied Biosystems, Foster City, CA, USA), following the manufacturer’s instructions. Gene expression was quantified by qPCR using the Maxima SYBR Green/ROX (2x) qPCR master mix (Thermo Scientific, Waltham, MA, USA). The 18S rRNA gene was used as an endogenous control. Relative expression levels were calculated using the ΔΔCt method [33]. The following primers were used for qPCR amplification: E6 Forward 5′-TTTCAGGACCCACAGGAGCGA-3′, E6 Reverse 5′-AGTCATATACCTCACGTCGCAGTA-3′; E7 Forward 5′-CAAGTGTGACTCTACGCTTCGG-3′; E7 Reverse 5′-TGTGCCCATTAACAGGTCTTCCAA-3′; PI3KC2A Forward 5′-TGTCGAGACTCTTGCCATTAC-3′; *PI3KC2A* Reverse 5′-GCTAGTGTCTTCTCCTCCAAAC-3′; 18S Forward 5′-AACCCGTTGAACCCCATT-3′; and 18S Reverse 5′-CCATCCAATCGGTAGTAGCG-3′.

### 2.4. Immunodetection Assay

A total of 500,000 cells were seeded in 60 mm culture dishes and, when appropriated, proteins were extracted with 200 µL of 2x Laemmli sample buffer (Bio-Rad, Hercules, CA, USA). Proteins samples were resolved on 6% and 15% SDS-PAGE gels and subsequently transferred onto a 0.22 µm nitrocellulose membrane (Bio-Rad, Hercules, CA, USA). Then, membranes were blocked with 10% skim milk in TBS-0.1% Tween 20 for 1 h at room temperature. Membranes were incubated with the following primary antibodies: anti-PI3K-C2α (1:500) (Abcam, Cambridge, UK), anti-p53 (1:1000) (Santa Cruz Biotechnology, Dallas, TX, USA), anti-HA-Tag (1:1000) (Cell Signaling, Danvers, MA, USA), anti-α-actinin (1:1000) (Santa Cruz Biotechnology, Dallas, TX, USA), anti-GFP (Santa Cruz Biotechnology, Dallas, TX, USA) and anti-α-tubulin (1:1000) (Santa Cruz Biotechnology, Dallas, TX, USA) overnight at 4 °C. After extensive washing with TBS-0.1% Tween 20, the membranes were incubated with HRP-conjugated anti-mouse (1:10,000) (Santa Cruz Biotechnology, Dallas, TX, USA) or anti-rabbit (1:10,000) (Santa Cruz Biotechnology, Dallas, TX, USA) secondary antibodies for 1 h at room temperature. Protein detection was performed by chemiluminescence using the Clarity Western ECL kit (Bio-Rad, Hercules, CA, USA), and signals were visualized using an iBright FL1500 imaging system (Invitrogen, Carlsbad, CA, USA). At least three independent experiments were performed to ensure reproducibility.

### 2.5. Cell Proliferation and Migration

A total of 8000 cells were seeded per well in 96-well plates and incubated for 72 h. Cell viability was assessed using a colorimetric assay based on tetrazolium compound [3-(4,5-dimethylthiazol-2-yl)-5-(3-carboxymethoxyphenyl)-2-(4-sulfophenyl)-2H-tetrazolium, inner salt; MTS] with the CellTiter 96 Aqueous One Solution Cell Proliferation Kit (Promega, Madison, WI, USA), following the manufacturer’s instructions. In parallel, crystal violet staining assays were performed to evaluate changes in cell density. Cells were first fixed with a 10% PBS/formaldehyde solution at room temperature for 30 min. After, cells were stained with 0.1% crystal violet in PBS for 15 min. After three washes, the dye was solubilized using 10% acetic acid/PBS solution and the absorbance was measured at 490 nm.

Cell migration was assessed using Transwell inserts with 8 µm pore size (Millipore, Darmstadt, Germany). A total of 150,000 cells per insert, transfected with either scrambled control RNA (Scr) or miR-218-5p mimic, were seeded into 24-well plates. Cells were incubated at 37 °C for 48 h and subsequently fixed with PBS/10% formaldehyde at room temperature for 30 min. Then, cells were stained with 0.1% crystal violet in PBS for 15 min. Finally, the dye was eluted with 10% acetic acid/PBS solution, and the absorbance was measured at 490 nm.

### 2.6. TCGA CC Patients Cohort and Data Acquisition

Transcriptomic, miRNA, and clinical data from patients with cervical squamous cell carcinoma (CESC) were obtained from The Cancer Genome Atlas (TCGA) data portal [34]. The mRNA sequencing counts, mature miRNA sequencing data, curated clinical annotations, and overall survival (OS) information were retrieved. Only primary tumor samples were included in the analyses. Samples were retained for analysis only if complete mRNA, miRNA, survival, and clinical data were available across all datasets.

### 2.7. Expression Preprocessing and Normalization

Transcriptomic and mature miRNA expressions were obtained from the TCGA data portal. Genes and miRNAs with minimal expression were filtered out by removing those with expression counts < 3 in all tumor samples, and retaining features with ≥3 counts in at least one tumor sample. Expression data were normalized using the trimmed mean of M-values (TMM) method implemented in edgeR [35,36], followed by conversion to counts per million (CPM) to ensure comparability across samples.

### 2.8. Survival Analysis and Statistical Modeling

OS was defined as the interval from diagnosis to death or last follow-up, with survival status coded as a binary event variable. Expression of hsa-miR-218-5p and *PIK3C2A* was dichotomized into high- and low-expression groups using the median as the cutoff. Kaplan–Meier survival curves were generated and compared using two-sided log-rank tests. Hazard ratios (HRs) and 95% confidence intervals (CIs) were estimated using Cox proportional hazards regression in R (version 4.5.2) using the *survival* (v. 3.8-3) [37,38] and *survminer* (v. 0.5.1) [39] packages. A multivariable Cox model including both miR-218-5p and *PIK3C2A* was fitted to assess their independent prognostic effects [40]. Statistical significance was defined as *p* < 0.05.

### 2.9. Statistical Analysis

All experiments were performed in independent biological triplicates, and data are presented as mean ± standard error of the mean (SEM). Student’s *t*-test was used to determine statistically significant changes in experimental conditions compared to the control, or, where indicated, in comparison with another experimental group. A two-way analysis of variance (ANOVA) was also used, followed by the Bonferroni multiple comparisons test. A value of *p* < 0.05 was considered statistically significant. Data analysis was performed using GraphPad Prism version 5.00 software for Windows.

## 3. Results

### 3.1. PIK3C2A and miR-218-5p Are Differentially Expressed in HPV-16-Positive and -Negative CC-Derived Cells

**Figure 1:**
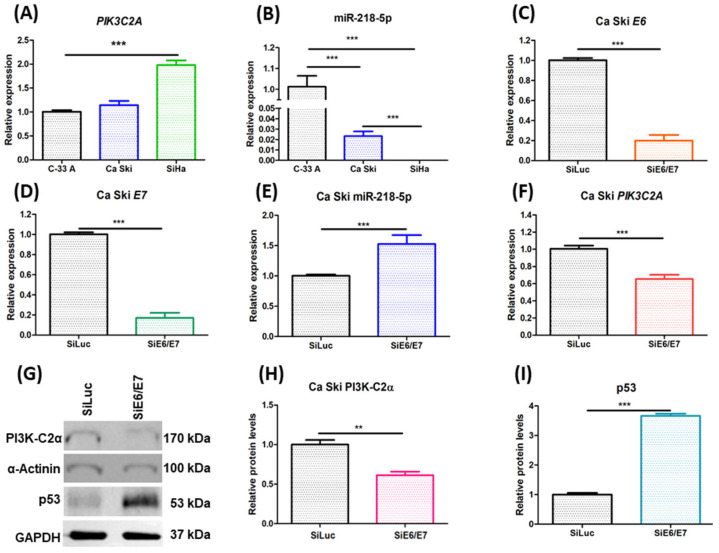
HPV-16 regulates miR-218-5p and *PIK3C2A* expression and reveals an inverse correlation in CC cells. (**A**) *PIK3C2A* expression is increased whereas (**B**) miR-218-5p expression is decreased in HPV-16-positive CC cell lines compared to HPV-negative cells. (**C**) Efficient knockdown of E6 and (**D**) E7 expression in Ca Ski cells transfected with siE6/E7 compared to siLuc control. (**E**) miR-218-5p is increased, while (**F**) *PIK3C2A* is decreased in E6/E7-silenced Ca Ski cell. (**G**) Representative immunoblot showing reduced PI3K-C2α protein levels and increased p53 levels upon E6/E7 silencing. (**H**,**I**) Densitometric analysis of PI3K-C2α and p53 protein levels, respectively, showing significant differences between siLuc control and siE6/E7-transfected Ca Ski cells. Each graph shows the mean ± standard error (SE) of three independent experiments. Statistical significance was assessed using Student’s *t*-test; statistically significant values are represented as ** *p* < 0.01 and *** *p* < 0.001.

To determine the role of HPV-16 in the expression of *PIK3C2A* and miR-218-5p, HPV-16 positive (SiHa and Ca Ski) and HPV-negative (C-33 A) CC cell lines were used. *PIK3C2A* expression was increased in SiHa cells, reaching a 1.98-fold change compared to C-33 A cells, whereas in Ca Ski cells the increase was more modest (1.14-fold) and not statistically significant (Figure 1A). In contrast, miR-218-5p expression was significantly reduced in Ca Ski cells, showing a 50-fold decrease relative to C-33 A cells, while in SiHa cells, miR-218-5p expression was undetectable (Figure 1B). These results strongly suggest that, in CC cells, HPV-16 downregulates miR-218-5p expression while promoting *PIK3C2A* expression.

Next, we assessed whether the HPV-16 E6 and E7 oncogenes affect the expression of miR-218 and *PIK3C2A*. For this purpose, Ca Ski cells were transfected with small interfering RNAs targeting E6 and E7 transcripts (siE6/E7). Efficient silencing of E6 and E7 expression was confirmed (Figure 1C,D). Notably, E6/E7 knockdown restored miR-218-5p expression by up to 1.52-fold increase (Figure 1E). In parallel, *PIK3C2A* expression showed a 1.54-fold decrease at the mRNA level (Figure 1F) and a 1.64-fold decrease at protein levels (Figure 1G,H), compared to Ca Ski cells transfected with a control interfering RNA (siLuc). As expected, p53 protein levels exhibited a 3.66-fold increase in E6/E7-silenced Ca Ski cells relative to the control group (siLuc) (Figure 1G,I).

### 3.2. HPV-16 E6 and E7 Oncoproteins Regulate the Expression of PIK3C2A and miR-218-5p

**Figure 2:**
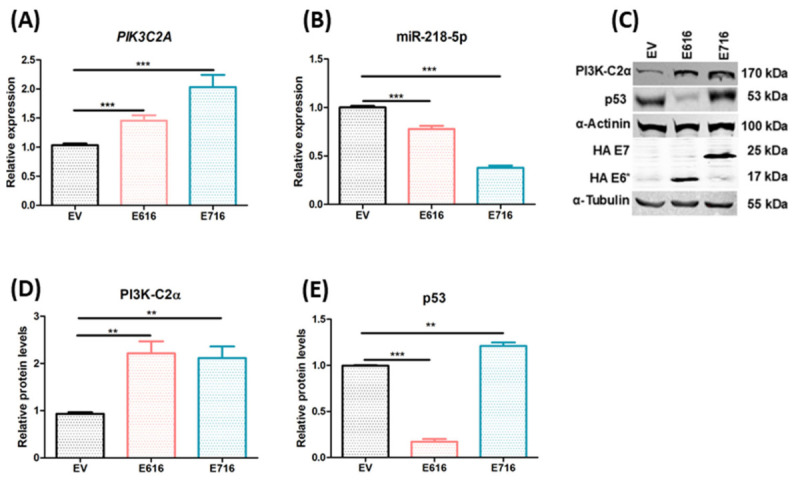
HPV-16 E6 and E7 oncoproteins increase *PIK3C2A* expression and protein levels while suppressing miR-218-5p expression. (**A**) HPV-16 E6 and E7 oncoproteins upregulated *PIK3C2A* mRNA expression, whereas (**B**) miR-218-5p expression is reduced in E6- and E7-expressing cells. (**C**) Inmunoblot showing increased PI3K-C2α protein levels in E6- and E7-expressing cells compared to control cells, along with a marked reduction in p53 protein levels in E6-expressing cells. (**D**,**E**) Densitometric analysis of PI3K-C2α and p53 protein level, respectively. Each graph shows the mean ± standard error (SE) of three independent experiments. Statistical significance was assessed using Student’s *t*-test; statistically significant values are represented as ** *p* < 0.01 and *** *p* < 0.001.

To further establish the causal role of HPV-16 oncoproteins E6 and E7 in the regulation of *PIK3C2A* and miR-218-5p expression, C-33 A cells stably transfected with E6 or E7 oncogenes were used. Expression of both oncoproteins significantly increased *PIK3C2A* expression, exhibiting 1.45- and 2-fold increases, respectively, compared to cells transfected with the empty vector (EV) (Figure 2A). In contrast, E6 and E7 significantly reduced miR-218-5p expression, with 1.30- and 2.70-fold decreases, respectively, relative to the EV cells (Figure 2B). Consistent with the gene expression data, PI3K-C2α protein levels were significantly increased in the presence of E6 and E7, showing 2.21- and 2.11-fold increases, respectively (Figure 2C,D). Furthermore, E7 protein and the alternatively spliced product E6* was detected in C-33 A cells. E6* is the most abundant protein product derived from E6 transcripts (Figure 2C); however, in E6-expressing cells, a substantial decrease in p53 levels was observed (Figure 2C,E). This suggests that although full-length E6 was not readily detectable, its functional impact on p53 is evident, possibly due to rapid proteasomal degradation of the E6/E6AP/p53 complex. These data support a direct role for E6 and E7 in regulating the *PIK3C2A*/miR-218-5p axis, ultimately affecting PI3K-C2α protein levels.

### 3.3. Increased Levels of miR-218-5p Decrease the Expression and Protein Levels of PIK3C2A in Cells Expressing HPV-16 E6 and E7 Oncogenes

**Figure 3:**
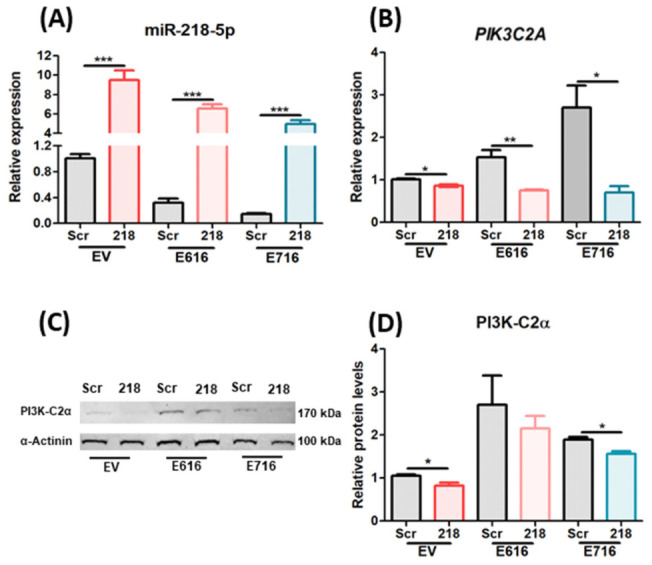
Restoration of miR-218-5p expression modulates *PIK3C2A* expression and protein levels in cells harboring HPV-16 E6 and E7 oncogenes. (**A**) miR-218-5p expression is increased following transfection with a miR-218-5p mimic (218) in EV, E6 and E7 cells, scrambled RNA sequence (Scr) was used as control. (**B**) *PIK3C2A* mRNA expression is decreased upon miR-218-5p overexpression compared to the corresponding Scr control. (**C**) Representative immunoblot showing reduced PI3K-C2α protein levels following miR-218-5p overexpression in both EV and E7 cells, with a more pronounced effect in E7 cells. (**D**) Densitometric analysis of PI3K-C2α protein levels showing significant differences between groups. Each graph shows the mean ± standard error (SE) of three independent experiments. Statistical significance was assessed using Student’s *t*-test; statistically significant values are represented as * *p* < 0.05, ** *p* < 0.01 and *** *p* < 0.001.

Given that *PIK3C2A* has been previously identified as a direct target of miR-218-5p, harboring two binding sites within its 3′UTR that promote transcript degradation [27], we next evaluated the effect of miR-218-5p on *PIK3C2A* expression. To this end, cells with stable expression of E6 or E7, as well as EV cells, were transfected with a miR-218-5p mimic, while a Scramble RNA sequence (Scr) was used as a control. As shown in Figure 3A, ectopic expression of miR-218-5p in EV cells resulted in a 9.51-fold increase relative to the Scr control. In cells expressing E6 or E7, miR-218-5p levels were restored, showing 13.8- and 16.33-fold increases, respectively, compared to their corresponding Scr controls (Figure 3A). Consistent with the proposed regulatory interaction, *PIK3C2A* expression exhibited a 1.16-fold decrease in EV cells following miR-218-5p overexpression. Notably, a more pronounced reduction was observed in E6- and E7-expressing cells, with 2.07- and 3.86-fold decreases, respectively, compared to their respective control groups (Figure 3B). Furthermore, restoration of miR-218-5p expression led to a 1.23-fold decrease in PI3K-C2α protein levels in EV cells and 1.77-fold decrease in E7-expressing cells relative to the Scr group (Figure 3C,D). No significant changes were observed in E6-expressing cells.

Taken together, these findings suggest that HPV-16 oncoproteins E6 and E7 promote *PIK3C2A* expression by negatively regulating miR-218-5p expression, as restoring miR-218-5p expression significantly reduces *PIK3C2A* expression levels, possibly through a direct interaction of miR-218-5p with the 3′UTR region of the *PIK3C2A* transcript.

### 3.4. miR-218-5p Overexpression Decreases Cell Proliferation and Migration Induced by HPV-16 Oncoproteins E6 and E7

**Figure 4:**
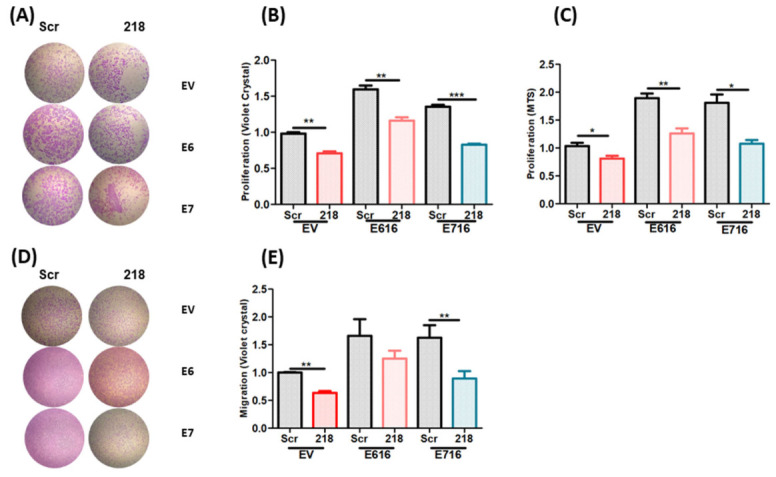
miR-218-5p decreased proliferation and migration of HPV-16 E6- and E7-expressing cells. (**A**,**B**) Ectopic expression of miR-218-5p in EV, E6- and E7-expressing cells reduces proliferation as determined by crystal violet assays, and (**C**) MTS assays compared to the corresponding scrambled RNA (Scr) controls. (**D**,**E**) miR-218-5p reduces migration capacity in EV and E7-expressing cells, with a more pronounced effect observed in E7 cells. Representative images were acquired with a 4x magnification objective using an optical microscope. Each graph shows the mean ± standard error (SE) of three independent experiments. Statistical significance was assessed using Student’s *t*-test; statistically significant values are represented as * *p* < 0.05, ** *p* < 0.01 and *** *p* < 0.001.

The impact of HPV-16 oncoproteins on cell proliferation and migration was assessed. Crystal violet assays showed that E6- and E7-expressing cells exhibited 1.59- and 1.35-fold increases in cell proliferation, respectively, compared to EV-transfected cells (Figure 4A,B). These findings were consistent with MTS assays, in which E6 and E7 induced 1.90- and 1.81-fold increases in proliferation, respectively (Figure 4C). In addition, both E6 and E7 enhanced cell migration, showing 1.64- and 1.62-fold increases, respectively, relative to EV control cells (Figure 4D,E).

We next investigated whether restoration of miR-218-5p expression could counteract these effects. Notably, miR-218-5p overexpression significantly reduced cell proliferation in E6- and E7-expressing cells, with 1.37- and 1.62-fold decreases, respectively, as deter-mined by crystal violet assays (Figure 4A,B), and 1.50- and 1.67-fold decreases, respectively, as measured by MTS (Figure 4C), compared to cells transfected with scrambled control (Scr). In EV-transfected cells, miR-218-5p overexpression also reduced proliferation, with a 1.42-fold decrease in crystal violet assays and a 1.23-fold decrease in MTS assays relative to the Scr control (Figure 4A–C).

Regarding cell migration, miR-218-5p overexpression significantly reduced migration in E7-expressing cells, showing a 1.82-fold decrease compared to the corresponding Scr control (Figure 4D,E). A reduction in migration was also observed in EV cells transfected with miR-218-5p, with a 1.58-fold decrease relative to the Scr group (Figure 4D,E). Although E6-expressing cells displayed a trend toward reduced migration following miR-218-5p restoration, this effect did not reach statistical significance (Figure 4D,E). Collectively, these results suggest that HPV-16 E7 protein plays a major role in promoting the proliferation and migration of CC cells, at least in part, by downregulating miR-218-5p.

### 3.5. Exogenous Expression of PI3KC2A Does Not Reverse miR-218-5p-Suppressed E7 Cell Proliferation

**Figure 5:**
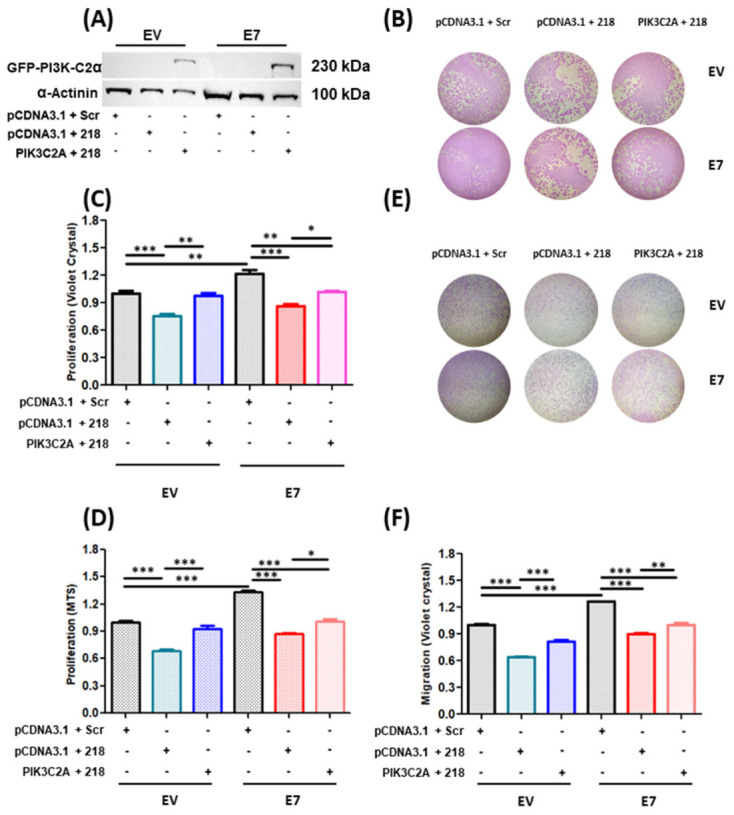
*PIK3C2A* partially rescues proliferation and migration in EV and E7 cells in the presence of miR-218-5p. (**A**) Immunodetection of ectopic PI3K-C2α expression detected by GFP-tag in EV- and E7-expressing cells. (**B**,**C**) miR-218-induced suppression of cell proliferation in EV and E7 cells is partially reversed by ectopic expression of *PIK3C2A*, as observed in crystal violet and (**D**) MTS assays. This effect is more pronounced in EV cells. (**E**,**F**) Similarly, miR-218-5p-mediated inhibition of cell migration is partially rescued upon *PIK3C2A* overexpression in both EV and E7 cells, with a stronger effect observed in EV cells. The graphs show the mean ± standard error (SE) of three independent experiments. Representative images were acquired with a 4x magnification objective using an optical microscope. Statistical significance was determined by a two-way ANOVA followed by the Bonferroni multiple comparisons test. Statistically significant values are represented as * *p* < 0.05, ** *p* < 0.01 and *** *p* < 0.001.

To determine the extent to which the miR-218-5p/*PIK3C2A* axis contributes to cell proliferation and migration, EV and E7-expressing cells were transfected with a plasmid encoding *PIK3C2A* fused to an N-terminal GFP tag (Figure 5A), to restore *PIK3C2A* expression suppressed by exogenous miR-218-5p. As shown in Figure 5, under control conditions (Scr and pCDNA 3.1 plasmid), E7 expressing cells exhibited 1.32- and 1.44-fold increases in proliferation compared to EV cells, as determined by crystal violet (Figure 5B,C) and MTS assays (Figure 5D), respectively. As expected, transfection with the miR-218-5p mimic significantly reduced cell proliferation, with 1.33- and 1.47-fold decreases in EV cells and 1.42- and 1.51-fold decreases in E7 cells, as observed in crystal violet (Figure 5B,C) and MTS assays (Figure 5D), respectively. Notably, ectopic expression of *PIK3C2A* partially reversed the miR-218-5p-induced suppression of proliferation in EV cells, with 1.29- and 1.35-fold increases, whereas in E7 cells, this recovery was modest, with only 1.18- and 1.14-fold increases, as observed in crystal violet assays (Figure 5B,C) and MTS assays (Figure 5D), respectively.

A similar trend was observed for cell migration; under control conditions (Scr and pCDNA 3.1), E7-expressing cells exhibited 1.32-fold increase compared to EV cells (Figure 5E,F). As expected, ectopic expression of miR-218-5p reduced migration in both EV and E7 cells, with 2.0- and 2.2-fold decreases, respectively. Furthermore, overexpression of *PIK3C2A* in miR-218-5p-transfected cells led to a 1.50-fold increase in EV cells, while only a 1.12-fold increase was observed in E7 cells (Figure 5E,F).

These findings indicate that, although E7 regulates miR-218-5p expression to influence cell proliferation and migration, *PIK3C2A* is unlikely to be the sole mediator of these effects. It is possible that E7 affects other transcripts, which are targets of miR-218-5p involved in cell proliferation and migration; therefore, additional pathways may contribute to the regulation of these biological processes, warranting further investigation.

### 3.6. PIK3C2A and miR-218-5p Expression Levels Are Associated with Clinical Outcome in Patients with CC

**Figure 6:**
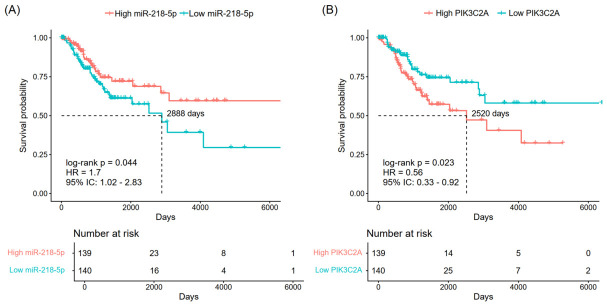
miR-218-5p and *PIK3C2A* expression are associated with poor prognosis in patients with CC. Kaplan–Meier survival curves show that (**A**) low miR-218-5p and (**B**) high *PIK3C2A* expression are associated with worse overall survival. Patients were stratified into high- (red lines) and low-expression (blue lines) levels according to the median expression levels. The dashed line indicates the 50% survival probability, and the time scale is shown in days. Survival analyses were performed using the Cox proportional hazards model implemented in the *survival* package in R with results presented as Kaplan–Meier survival curves.

Finally, to evaluate the potential clinical relevance of miR-218-5p and *PIK3C2A* ex-pression in CC prognosis, OS analyses were performed using TCGA transcriptomic data. Patients were stratified into high- and low-expression groups for each transcript, and only cases with both mRNA and miRNA sequencing data were included. Consistent with our experiment findings, patients with low miR-218-5p expression exhibited significantly worse OS (HR = 1.7, 95% CI: 1.02–2.83) (Figure 6A). In contrast, low *PIK3C2A* expression was significantly associated with improved OS, showing a protective effect (HR = 0.56, 95% CI: 0.33–0.92) (Figure 6B).

Taken together, these findings suggest that combined assessment of *PIK3C2A* and miR-218-5p may serve as potential prognostic and stratification tool in CC patients.

## 4. Discussion

Epigenetic regulatory mechanisms have been widely implicated in cancer progression, particularly through alterations in the expression of oncogenes and tumor suppressor genes, which contribute to the malignant phenotype. In this context, microRNAs (miRNAs) have emerged as key regulators of diverse cellular processes, including apoptosis, cell cycle progression, metastasis, invasion, and chemo- and radioresistance [41]. miRNAs are short, 18–25 nucleotide-long, non-coding RNA molecules that regulate gene expression by reducing mRNA stability, promoting transcript degradation, and inhibiting translation [42].

During CC development, HPV oncoproteins E6 and E7 have been shown to alter miRNA expression profiles, thereby contributing to the acquisition of cervical cancer hallmarks [43,44,45]. Expression profiling studies have identified multiple dysregulated miRNAs in HPV-positive cancer-derived cell lines. Among these, the immature miR-218 and the mature strand miR-218-5p are consistently downregulated in HPV-positive cell lines [46,47]. Moreover, reduced miR-218 and specifically miR-218-5p expression has been reported in premalignant lesions and CC tissues compared to normal cervical epithelium [46,47,48], with progressively lower levels observed as the disease advances [30]. Furthermore, it had been shown that miR-218-1 and -2 promoters are hypermethylated in cervical cancer samples compared to premalignant cervical lesions, which correlates with lower miR-218 expression during tumor progression [29]. Bioinformatic pathway enrichment analyses further suggest that suppression of miR-218-5p in CC is associated with the deregulation of genes linked to key oncogenic signaling pathways including PI3K/Akt and mTOR signaling pathways [30,46].

Previous studies have reported decreased miR-218 expression in CC-derived cell lines, including HeLa, SiHa, Ca Ski and C-33 A, compared to primary cervical epithelial cells or normal tissue [47,48,49]. However, these analyses did not distinguish between the mature -5p and -3p strands of miR-218. In agreement, reduced miR-218-5p expression has been observed in C-33 A and Ca Ski CC cells compared to immortalized HaCaT cells; however, no significant differences were reported between HPV-positive and HPV-negative CC cell lines [28]. In contrast, other studies have shown decreased miR-218-5p expression in Ca Ski cells relative to C-33 A cells [30]. Consistent with these findings, our results demonstrate that miR-218-5p expression is significantly reduced in HPV-positive cells compared to HPV-negative cells.

Functional studies have investigated the role of miR-218-5p in cellular processes associated with CC and have identified several relevant molecular targets. For instance, overexpression of miR-218-5p has been shown to suppress the expression of *RUNX2*, a validated target gene [30]. Similarly, the *IDO1* (Indoleamine-2,3-dioxygenase 1) transcript contains miR-218 binding sites, leading to its degradation [49]. In addition, miR-218-5p overexpression significantly reduces *NACC1* expression in CC cells, consistent with the presence of miR-218-5p binding elements within its transcript [28]. miR-218 has also been reported to regulate *GLI3* expression through direct interaction with its mRNA [48]. Furthermore, a combinatorial miRNA overexpression study involving miR-23b-3p, miR-218-5p, and miR-124-3p in Ca Ski and C-33 A cells demonstrated that downregulated target genes were enriched in pathways related to proliferation and migration, including PI3K/Akt signaling pathway. Notably, among the genes affected was *PIK3C2A*, whose elevated expression has been associated with poor prognosis in patients with CC [26]. Similarly, low expression of miR-218-5p has also been linked to a worse clinical outcome in these patients [30].

Our results revealed that *PIK3C2A* expression is significantly increased in HPV-positive cells. Moreover, a transcriptomic analysis identified *PIK3C2A* as one of the genes specifically deregulated by HPV-16 E6 and E7 [31]. In this context, we found that miR-218-5p expression is markedly reduced in HPV-16 E6- and E7-expressing cells and exhibits a strong inverse correlation with *PIK3C2A* expression. In agreement, miR-218 expression was decreased in osteosarcoma cells expressing HPV-16 E6 and E7 oncogenes. Also, increased miR-218 expression was detected in keratinocytes harboring tandemly integrated copies of the HPV-16 genome following E6/E7 silencing; meanwhile, normal oral keratinocytes (NOKs) expressing HPV-16 E6 exhibited lower miR-218 expression levels. Importantly, this study assessed immature miR-218 expression and did not distinguish between mature strands. This is relevant because the biological activity and target specificity of miRNAs are highly dependent on the mature strand involved [47].

Furthermore, it is important to note that C-33 A cells harbor a p53 mutation at codon 273, resulting in the substitution of arginine by cysteine and the loss of its canonical DNA-binding transcriptional activity [50]. Nevertheless, non-transcriptional functions of mutant p53, including those associated with the cytoplasm and mitochondria, may remain partially preserved [51,52]. In our model, p53 was efficiently degraded by E6, indicating that this oncoprotein is functionally active and capable of suppressing residual p53 activities in C-33 A cells. To our knowledge, neither miR-218 nor *PIK3C2A* has been identified as a direct transcriptional target of p53. Therefore, the alterations observed in the miR-218-5p/*PIK3C2A* axis are unlikely to be a direct consequence of canonical p53-dependent transcriptional activity. As discussed above, these oncoproteins have been shown to downregulate miR-218 expression in both transformed and non-transformed cellular models, suggesting that repression of miR-218 constitutes a broader HPV-driven event rather than a consequence of a specific p53 mutational background. However, it would be worthwhile to investigate in the future whether wild-type p53 or its distinct mutant variants can modulate this regulatory axis in HPV-associated cancer progression.

PI3K-C2α contributes to cell survival through regulation of the PI3K/Akt/mTOR signaling pathway. In addition, during mitosis, PI3K-C2α localizes to the mitotic spindle, where it contributes to spindle stability and proper chromosome segregation. In a chondrosarcoma model, miR-218-5p was shown to directly regulate *PIK3C2A* mRNA by interacting with two binding sites located at positions 78–485 and 2184–2190 nt within the 3′UTR region. Mechanistically, reduced expression of miR-218-5p was found to promote *PIK3C2A* expression and activation of downstream effectors of the PI3K/Akt/mTOR signaling pathway, including Akt, mTOR, S6, and 4EBP1, thereby affecting cell viability [27]. Concordantly, our results indicate that ectopic expression of miR-218-5p reduces both *PIK3C2A* and protein levels with a more pronounced effect observed in cells expressing E6 and E7, indicating that E6 and E7 contribute to the upregulation of *PIK3C2A*, at least in part, through suppression of miR-218-5p expression. Further studies will be required to demonstrate the involvement of miR-218-5p in the PI3K/Akt/mTOR signaling pathway in the presence of the HPV-16 oncoproteins E6 and E7.

Functionally, miR-218-5p exerts tumor-suppressive effects in CC models. Its overexpression has been associated with reduced cell proliferation and increased apoptosis in C-33 A and Ca Ski cells [28]. Likewise, miR-218 overexpression decreases cell viability, promotes apoptosis, and downregulates genes involved in immune evasion, including TGF-β, VEGF, IL-6, and COX-2, while also inhibiting the JAK/STAT signaling pathway [49]. In SiHa cells, miR-218 overexpression markedly suppresses proliferation and induces cell cycle arrest at the G0/G1 phase, accompanied by increased levels of cleaved caspase-3 and PARP, and reduced expression of cyclins D1 and B1 [48]. Our results reveal that restoration of miR-218-5p attenuates HPV-16 oncogene-induced proliferation E6 and E7, and significantly reduces migration only in E7-expressing cells. This suggests that miR-218-5p is a key element dysregulated by E7 for the maintenance of cell proliferation and migration.

As mentioned above, miR-218-5p negatively regulates *PIK3C2A* mRNA, thereby reducing the activation of components of the PI3K/Akt/mTOR signaling pathway in chondrosarcoma model [27]. It was demonstrated that ectopic expression of *PIK3C2A* can restore PI3K/Akt/mTOR signaling pathway activity despite miR-218-5p overexpression, as *PIK3C2A* ectopic mRNA lacks the native 3′UTR and is therefore not susceptible to miRNA-mediated degradation [27]. However, our results show that in cells expressing E7, ectopic expression of miR-218-5p markedly decreases proliferation and migration, effects that are not counteracted by ectopic expression of *PIK3C2A*. These findings suggest that, while *PIK3C2A* is a relevant downstream target of miR-218-5p, it is unlikely to be the sole mediator of its effects in the context of E7 expression. Instead, additional E7-regulated genes may contribute to maintaining the proliferative and migratory phenotype. Supporting this notion, transcriptomic studies have shown that E7 induces the expression of genes such as *NAAC1* and *SIK2* [31], which are involved in the proliferation and migration and have been proposed as potential targets of miR-218-5p [25]. Taken together, these results indicate that, in E7-expressing cells, miR-218-5p likely exerts its tumor-suppressive effects through the coordinated regulation of multiple targets, rather than exclusively through *PIK3C2A*.

Finally, our OS analysis revealed that low miR-218-5p expression and high *PIK3C2A* expression were both associated with poorer OS. These findings are consistent with previously published reports [26,30] and support the potential of miR-218-5p and *PIK3C2A* as prognostic biomarkers in CC.

## 5. Conclusions

Our findings, performed in the C-33 A cell line stably transfected with the HPV-16 E6 and E7 oncogenes, indicate that these oncoproteins contribute to the downregulation of miR-218-5p, which is associated with increased *PIK3C2A* expression and enhanced proliferation and migration of CC cells. We demonstrated that miR-218-5p regulates *PIK3C2A* expression and provided functional evidence that this regulatory axis contributes, although not exclusively, to the control of the tumor-associated phenotypes, particularly in the context of E7 expression. Our results also raise the possibility that modulation of the miR-218-5p/*PIK3C2A* axis may represent a conserved mechanism among high-risk HPV types, although further studies are required to validate this hypothesis. Importantly, our bioinformatic analyses support the clinical significance of this regulatory network, where low miR-218-5p expression and high *PIK3C2A* levels are associated with poorer OS in patients with CC. Together, these findings highlight the miR-218-5p/*PIK3C2A* axis as a novel regulatory pathway involved in HPV-driven cervical progression and suggest its potential utility as a prognostic biomarker and therapeutic target.

## Data Availability

The original contributions presented in this study are included in the article. Further inquiries can be directed to the corresponding authors.

## Abbreviations

The following abbreviations are used in this manuscript:

HPV	Human papillomavirus
PIK3C2A	Phosphatidylinositol-4-Phosphate 3-Kinase Catalytic Subunit Type 2 Alpha
TCGA	The Cancer Genome Atlas
E6AP	Human Papilloma Virus E6-Associated Protein
p53	Tumor Protein P53
pRB	Retinoblastoma Protein
PI3K	Phosphatidylinositol 3-kinase
PI(2,5)P2	Phosphatidylinositol-4,5-bisphosphate
PI(3,4,5)P3	Phosphatidylinositol-3,4,5-trisphosphate
PI3K-C2α	Phosphatidylinositol-4-Phosphate 3-Kinase Catalytic Subunit Type 2 Alpha protein
PI3K-C2β	Phosphatidylinositol-4-Phosphate 3-Kinase Catalytic Subunit Type 2 Beta protein
PI3K-C2γ	Phosphatidylinositol-4-Phosphate 3-Kinase Catalytic Subunit Type 2 Gamma protein
PI3P	Phosphatidylinositol 3-phosphate
PI(3,4,5)P3	Phosphatidylinositol (3,4,5)-trisphosphate
EGFR	Epidermal Growth Factor Receptor
PDGFR	Platelet Derived Growth Factor Receptor
Rab11	Ras-Related Protein Rab-11A
RhoA	Ras Homolog Family Member A
Akt	AKT Serine/Threonine Kinase
HIF-1α	Hypoxia Inducible Factor 1 Subunit Alpha
VEGFA	Vascular Endothelial Growth Factor A
mTOR	Mechanistic Target Of Rapamycin Kinase
ATCC	American Type Culture Collection
HA	Hemagglutinin Tag
DMEM	Dulbecco’s Modified Eagle’s Medium
RPMI	Roswell Park Memorial Institute Medium
siRNA	Small Interfering RNA
rRNA	Ribosomal RNA
mRNA	Messenger RNA
CESC	Cervical Squamous Cell Carcinoma
OS	Overall Survival
CPM	Counts Per Million
TMM	Trimmed Mean of M-values
HR	Hazard Ratio
CI	Confidence Intervals
SEM	Standard Error of the Mean
UTR	Untranslated Region
tsmiRs	Tumor Suppressor miRNAs
oncomiRs	Oncogenic miRNAs
IDO1	Indoleamine-2,3-dioxygenase 1
NACC1	Nucleus Accumbens Associated 1
RUNX2	RUNX Family Transcription Factor 2
TGF-β	Transforming Growth Factor Beta
VEGF	Vascular Endothelial Growth Factor
IL-6	Interleukin 6
COX-2	Cyclooxygenase 2
JAK	Janus Kinase
STAT	Signal Transducer And Activator Of Transcription
PARP	Poly(ADP-Ribose) Polymerase
SIK2	Salt Inducible Kinase 2

## References

[1] Cancer Today. https://gco.iarc.who.int/today/en.

[2] Della Fera A.N., Warburton A., Coursey T.L., Khurana S., McBride A.A. (2021). Persistent Human Papillomavirus Infection. Viruses.

[3] Schiffman M., Doorbar J., Wentzensen N., De Sanjosé S., Fakhry C., Monk B.J., Stanley M.A., Franceschi S. (2016). Carcinogenic Human Papillomavirus Infection. Nat. Rev. Dis. Prim..

[4] Bodily J., Laimins L.A. (2011). Persistence of Human Papillomavirus Infection: Keys to Malignant Progression. Trends Microbiol..

[5] de Martel C., Plummer M., Vignat J., Franceschi S. (2017). Worldwide Burden of Cancer Attributable to HPV by Site, Country and HPV Type. Int. J. Cancer.

[6] Pal A., Kundu R. (2020). Human Papillomavirus E6 and E7: The Cervical Cancer Hallmarks and Targets for Therapy. Front. Microbiol..

[7] Vallejo-Ruiz V., Gutiérrez-Xicotencatl L., Medina-Contreras O., Lizano M. (2024). Molecular Aspects of Cervical Cancer: A Pathogenesis Update. Front. Oncol..

[8] Li S., Hong X., Wei Z., Xie M., Li W., Liu G., Guo H., Yang J., Wei W., Zhang S. (2019). Ubiquitination of the HPV Oncoprotein E6 Is Critical for E6/E6AP-Mediated P53 Degradation. Front. Microbiol..

[9] Huh K., Zhou X., Hayakawa H., Cho J.-Y., Libermann T.A., Jin J., Wade Harper J., Munger K. (2007). Human Papillomavirus Type 16 E7 Oncoprotein Associates with the Cullin 2 Ubiquitin Ligase Complex, Which Contributes to Degradation of the Retinoblastoma Tumor Suppressor. J. Virol..

[10] Estêvão D., Costa N.R., Gil da Costa R.M., Medeiros R. (2019). Hallmarks of HPV Carcinogenesis: The Role of E6, E7 and E5 Oncoproteins in Cellular Malignancy. Biochim. Biophys. Acta Gene Regul. Mech..

[11] Bello J.O.M., Nieva L.O., Paredes A.C., Gonzalez A.M.F., Zavaleta L.R., Lizano M. (2015). Regulation of the Wnt/β-Catenin Signaling Pathway by Human Papillomavirus E6 and E7 Oncoproteins. Viruses.

[12] Muñoz-Bello J.O., Olmedo-Nieva L., Castro-Muñoz L.J., Manzo-Merino J., Contreras-Paredes A., González-Espinosa C., López-Saavedra A., Lizano M. (2018). HPV-18 E6 Oncoprotein and Its Spliced Isoform E6*I Regulate the Wnt/β-Catenin Cell Signaling Pathway through the TCF-4 Transcriptional Factor. Int. J. Mol. Sci..

[13] Weijzen S., Zlobin A., Braid M., Miele L., Kast W.M. (2003). HPV16 E6 and E7 Oncoproteins Regulate Notch-1 Expression and Cooperate to Induce Transformation. J. Cell. Physiol..

[14] Zhang L., Wu J., Ling M.T., Zhao L., Zhao K.N. (2015). The Role of the PI3K/Akt/MTOR Signalling Pathway in Human Cancers Induced by Infection with Human Papillomaviruses. Mol. Cancer.

[15] Vats A., Trejo-Cerro O., Thomas M., Banks L. (2021). Human Papillomavirus E6 and E7: What Remains?. Tumour Virus Res..

[16] He Y., Sun M.M., Zhang G.G., Yang J., Chen K.S., Xu W.W., Li B. (2021). Targeting PI3K/Akt Signal Transduction for Cancer Therapy. Signal Transduct. Target. Ther..

[17] Manzo-Merino J., Contreras-Paredes A., Vázquez-Ulloa E., Rocha-Zavaleta L., Fuentes-Gonzalez A.M., Lizano M. (2014). The Role of Signaling Pathways in Cervical Cancer and Molecular Therapeutic Targets. Arch. Med. Res..

[18] Martini M., Ciraolo E., Gulluni F., Hirsch E. (2013). Targeting PI3K in Cancer: Any Good News?. Front. Oncol..

[19] Gaidarov I., Smith M.E.K., Domin J., Keen J.H. (2001). The Class II Phosphoinositide 3-Kinase C2alpha Is Activated by Clathrin and Regulates Clathrin-Mediated Membrane Trafficking. Mol. Cell.

[20] Mazza S., Maffucci T. (2011). Class II Phosphoinositide 3-Kinase C2alpha: What We Learned so Far. Int. J. Biochem. Mol. Biol..

[21] Gulluni F., De Santis M.C., Margaria J.P., Martini M., Hirsch E. (2019). Class II PI3K Functions in Cell Biology and Disease. Trends Cell Biol..

[22] Chai Z.T., Kong J., Zhu X.D., Zhang Y.Y., Lu L., Zhou J.M., Wang L.R., Zhang K.Z., Zhang Q.B., Ao J.Y. (2013). MicroRNA-26a Inhibits Angiogenesis by down-Regulating VEGFA through the PIK3C2α/Akt/HIF-1α Pathway in Hepatocellular Carcinoma. PLoS ONE.

[23] Ng S.K.L., Neo S.Y., Yap Y.W., Karuturi R.K.M., Loh E.S.L., Liau K.H., Ren E.C. (2009). Ablation of Phosphoinositide-3-Kinase Class II Alpha Suppresses Hepatoma Cell Proliferation. Biochem. Biophys. Res. Commun..

[24] Gulluni F., Martini M., De Santis M.C., Campa C.C., Ghigo A., Margaria J.P., Ciraolo E., Franco I., Ala U., Annaratone L. (2017). Mitotic Spindle Assembly and Genomic Stability in Breast Cancer Require PI3K-C2α Scaffolding Function. Cancer Cell.

[25] Romero-López M.J., Jiménez-Wences H., Cruz-De la Rosa M.I., Román-Fernández I.V., Fernández-Tilapa G. (2022). MiR-23b-3p, MiR-124-3p and MiR-218-5p Synergistic or Additive Effects on Cellular Processes That Modulate Cervical Cancer Progression? A Molecular Balance That Needs Attention. Int. J. Mol. Sci..

[26] Romero-López M.J., Jiménez-Wences H., Nuñez-Martínez H.N., Cruz-De la Rosa M.I., Alarcón-Millán J., Fernández-Tilapa G. (2025). Overexpression of MiR-23b-3p+miR-218-5p+miR-124-3p Differentially Modifies the Transcriptome of C-33A and CaSki Cells and the Regulation of Cellular Processes Involved in the Progression of Cervical Cancer. Comput. Biol. Med..

[27] Lu J., Ji M.L., Zhang X.J., Shi P.L., Wu H., Wang C., Im H.J. (2017). MicroRNA-218-5p as a Potential Target for the Treatment of Human Osteoarthritis. Mol. Ther..

[28] Romero-López M.J., Jiménez-Wences H., Cruz-De La Rosa M.I., Alarcón-Millán J., Mendoza-Catalán M.Á., Ortiz-Sánchez E., Tinajero-Rodríguez J.M., Hernández-Sotelo D., Valente-Niño G.W., Martínez-Carrillo D.N. (2024). MiR-218-5p, MiR-124-3p and MiR-23b-3p Act Synergistically to Modulate the Expression of NACC1, Proliferation, and Apoptosis in C-33A and CaSki Cells. Noncoding RNA Res..

[29] Jiménez-Wences H., Martínez-Carrillo D.N., Peralta-Zaragoza O., Campos-Viguri G.E., Hernández-Sotelo D., Jiménez-López M.A., Muñoz-Camacho J.G., Garzón-Barrientos V.H., Illades-Aguiar B., Fernández-Tilapa G. (2016). Methylation and Expression of MiRNAs in Precancerous Lesions and Cervical Cancer with HPV16 Infection. Oncol. Rep..

[30] Cruz-De la Rosa M.I., Jiménez-Wences H., Alarcón-Millán J., Romero-López M.J., Castañón-Sánchez C.A., Salmerón-Bárcenas E.G., Fernández-Tilapa G. (2022). MiR-218-5p/RUNX2 Axis Positively Regulates Proliferation and Is Associated with Poor Prognosis in Cervical Cancer. Int. J. Mol. Sci..

[31] Olmedo-Nieva L., Muñoz-Bello J.O., Martínez-Ramírez I., Martínez-Gutiérrez A.D., Ortiz-Pedraza Y., González-Espinosa C., Madrid-Marina V., Torres-Poveda K., Bahena-Roman M., Lizano M. (2022). RIPOR_2_ Expression Decreased by HPV-16 E6 and E7 Oncoproteins: An Opportunity in the Search for Prognostic Biomarkers in Cervical Cancer. Cells.

[32] Posor Y., Eichhorn-Gruenig M., Puchkov D., Schöneberg J., Ullrich A., Lampe A., Müller R., Zarbakhsh S., Gulluni F., Hirsch E. (2013). Spatiotemporal Control of Endocytosis by Phosphatidylinositol-3,4-Bisphosphate. Nature.

[33] Livak K.J., Schmittgen T.D. (2001). Analysis of Relative Gene Expression Data Using Real-Time Quantitative PCR and the 2(-Delta Delta C(T)) Method. Methods.

[34] GDC Data Portal Homepage. https://portal.gdc.cancer.gov/.

[35] Robinson M.D., Oshlack A. (2010). A Scaling Normalization Method for Differential Expression Analysis of RNA-Seq Data. Genome Biol..

[36] Robinson M.D., McCarthy D.J., Smyth G.K. (2010). EdgeR: A Bioconductor Package for Differential Expression Analysis of Digital Gene Expression Data. Bioinformatics.

[37] Therneau T.M. (2026). Survival Analysis [R Package Survival Version 3.8-6]. CRAN: Contributed Packages.

[38] Therneau T.M. CRAN: Package Survival. https://cran.r-project.org/web/packages/survival/index.html.

[39] Alboukadel K., Marcin K., Przemyslaw B. Drawing Survival Curves Using Ggplot2 • Survminer. https://rpkgs.datanovia.com/survminer/index.html.

[40] Therneau T.M., Grambsch P.M. (2000). Modeling Survival Data: Extending the Cox Model.

[41] Causin R.L., de Freitas A.J.A., Filho C.M.T.H., Dos Reis R., Reis R.M., Marques M.M.C. (2021). A Systematic Review of MicroRNAs Involved in Cervical Cancer Progression. Cells.

[42] Svoronos A.A., Engelman D.M., Slack F.J. (2016). OncomiR or Tumor Suppressor? The Duplicity of MicroRNAs in Cancer. Cancer Res..

[43] Gómez-Gómez Y., Organista-Nava J., Gariglio P. (2013). Deregulation of the MiRNAs Expression in Cervical Cancer: Human Papillomavirus Implications. BioMed Res. Int..

[44] Zheng Z.M., Wang X. (2011). Regulation of Cellular MiRNA Expression by Human Papillomaviruses. Biochim. Biophys. Acta.

[45] Rao Q., Zhou H., Peng Y., Li J., Lin Z. (2012). Aberrant MicroRNA Expression in Human Cervical Carcinomas. Med. Oncol..

[46] Nair V.B., Manasa V.G., Sinto M.S., Jayasree K., James F.V., Kannan S. (2018). Differential Expression of MicroRNAs in Uterine Cervical Cancer and Its Implications in Carcinogenesis; An Integrative Approach. Int. J. Gynecol. Cancer.

[47] Martinez I., Gardiner A.S., Board K.F., Monzon F.A., Edwards R.P., Khan S.A. (2008). Human Papillomavirus Type 16 Reduces the Expression of MicroRNA-218 in Cervical Carcinoma Cells. Oncogene.

[48] Zhang J., Li S., Li Y., Liu H., Zhang Y., Zhang Q. (2018). MiRNA-218 Regulates the Proliferation and Apoptosis of Cervical Cancer Cells via Targeting Gli3. Exp. Ther. Med..

[49] Zhu L., Tu H., Liang Y., Tang D. (2018). MiR-218 Produces Anti-Tumor Effects on Cervical Cancer Cells In Vitro. World J. Surg. Oncol..

[50] Crook T., Wrede D., Vousden K.H. (1991). P53 Point Mutation in HPV Negative Human Cervical Carcinoma Cell Lines. Oncogene.

[51] Green D.R., Kroemer G. (2009). Cytoplasmic Functions of the Tumour Suppressor P53. Nature.

[52] Marchenko N.D., Moll U.M. (2014). Mitochondrial Death Functions of P53. Mol. Cell. Oncol..

